# Integration of smoking cessation into standard treatment for patients receiving opioid agonist therapy who are smoking tobacco: protocol for a randomised controlled trial (ATLAS4LAR)

**DOI:** 10.1186/s13063-022-06560-x

**Published:** 2022-08-17

**Authors:** Karl Trygve Druckrey-Fiskaaen, Einar Furulund, Jan Tore Daltveit, Jørn Henrik Vold, Torgeir Gilje Lid, Tesfaye Madebo, Lars Thore Fadnes, for the ATLAS4LAR Study Group, Vibeke Bråthen Buljovcic, Trude Fondenes, Per Gundersen, Siv-Elin Leirvåg Carlsen, Mette Hegland Nordbotn, Marianne Cook Pierron, Christine Sundal, Jørn Henrik Vold, Maren Borsheim Bergsaker, Eivin Dahl, Tone Lise Eielsen, Torhild Fiskå, Eirik Holder, Tine Selmer Cruickshank, Ewa Joanna Wilk

**Affiliations:** 1 Bergen Addiction Research, Department of Addiction Medicine, Haukeland University Hospital, Bergen, Norway; 2 Department of Global Public Health and Primary Care, University of Bergen, Bergen, Norway; 3 Centre for Alcohol and Drug Research, Stavanger University Hospital, Stavanger, Norway; 4 Division of Psychiatry, Haukeland University Hospital, Bergen, Norway; 5 Department of Respiratory Medicine, Stavanger University Hospital, Stavanger, Norway

**Keywords:** Smoking cessation, Nicotine replacement, Opiate substitution treatment, Behavioural intervention, Substance abuse treatment centres

## Abstract

**Background:**

About 85% of patients receiving opioid agonist therapy (OAT) for opioid dependence are smoking tobacco. Although smoke-related pulmonary diseases are significant contributors to morbidity and mortality, few smoking cessation interventions are evaluated within this group, and few OAT patients are offered smoking cessation as an integrated part of their addiction treatment. This study protocol describes an integrated smoking cessation intervention aimed at patients receiving OAT and smoking tobacco.

**Methods:**

This is a multicentre, randomised controlled clinical trial that will recruit 266 daily tobacco smoking patients receiving OAT in OAT outpatient clinics in Bergen and Stavanger, Norway. The patients randomised for the intervention arm will be offered smoking cessation therapy consisting of weekly brief behavioural interventions and prescription-free nicotine replacement products. In the control arm, patients will receive standard care without any added interventions related to smoking cessation.

The smoking cessation intervention includes psychoeducational techniques with components from motivational interviewing, and nicotine replacement products such as nicotine lozenges, patches, and chewing gum. The duration of the intervention is 16 weeks, with the option of extending it by a further 8 weeks. The main outcomes are measured at 16 weeks after initiation of the intervention, and sustained effects are evaluated 1 year after intervention initiation.

The primary outcome is smoking cessation verified by carbon monoxide (CO) levels or at least a 50% reduction in the number of cigarettes smoked. Secondary outcomes are changes in psychological well-being, biochemical inflammation markers, changes in physical health, quality of life, and fatigue.

**Discussion:**

Integration of other treatments to standard OAT care improves adherence and completion rates providing another rationale for integrated smoking cessation treatment. Thus, if integrated smoking cessation treatment is superior to standard care, this trial provides important information on further scale-up.

**Trial registration:**

ClinicalTrials.gov NCT05290025. Registered on 22 March 2022

## Administrative information

Note: The numbers in curly brackets in this protocol refer to SPIRIT checklist item numbers. The order of the items has been modified to group similar items (see http://www.equator-network.org/reporting-guidelines/spirit-2013-statement-defining-standard-protocol-items-for-clinical-trials/).

**Table:** 

Title {1}	Integration of smoking cessation into standard treatment for patients receiving opioid agonist therapy who are smoking tobacco: protocol for a randomised controlled trial (ATLAS4LAR)
Trial registration {2a and 2b}.	Registered in ClinicalTrials.gov NCT05290025, Date of registry 22 March 2022
Protocol version {3}	Version 2, 14.07.2022 Corrections made to SPIRIT ITEMS: 5c, 8, 26b, 16a, 33, 20c, 25 and 32. In addition, typographical errors have been corrected.
Funding {4}	The study was funded by Western Norway Regional Health Authority («Strategiske forskningsmidler» through ATLAS4LAR-project) with Department of Addiction Medicine, Haukeland University Hospital as responsible institution.
Author details {5a}	^1^ Bergen Addiction Research, Department of Addiction Medicine, Haukeland University Hospital, Bergen, Norway ^2^ Department of Global Public Health and Primary Care, University of Bergen, Norway ^3^ Centre for Alcohol and Drug Research, Stavanger University Hospital, Stavanger, Norway ^4^ Division of Psychiatry, Haukeland University Hospital, Bergen, Norway. ^5^ Department of Respiratory Medicine, Stavanger University Hospital, Stavanger, Norway ^6^ List of Members of the ATLAS4LAR Study Group (see acknowledgement)
Name and contact information for the trial sponsor {5b}	Department of Addiction Medicine, Haukeland University Hospital, director Christian Ohldieck, Post box 1400, 5021 Bergen. Christian.ohldieck@helse-bergen.no
Role of sponsor {5c}	The sponsor had no role in the study design and will have no role in data collection and analysis, decision to publish or preparation of the manuscript. The sponsor does and will not have ultimate authority over any of these activities.

## Introduction

### Background and rationale {6a}

Lung disease and tobacco smoking contribute to a high burden of disease [1]. Tobacco smoking alone leads to seven million deaths yearly and loss of 171 million disability-adjusted life-years worldwide [1]. Chronic obstructive lung disease (COPD), airway infections, and cardiovascular disease are highly linked to tobacco smoking and are the most important reasons for the global tobacco-related disease burden. Indeed, COPD alone causes three million deaths and loss of approximately 50 million disability-adjusted life-years yearly [2]. Although smoking cessation provides the largest mortality reduction, reducing smoking intensity also diminishes mortality [3, 4], providing a more feasible approach for some populations. Persons with opioid dependence syndrome experience higher morbidity and lower quality of life and have a substantially shorter expected lifespan than the general population [5–9]. Among patients with opioid dependence receiving opioid agonist therapy (OAT), nearly 85% smoke tobacco [10]. The prevalence of lung diseases is as high as 63% in autopsy samples of OAT patients [11], whereas 21% of patients on methadone as OAT medication in a primary care setting had diagnosis of COPD, asthma, or both [12]. Thus, ceasing tobacco smoking is likely to provide a significant decrease in morbidity and mortality among opioid-dependent patients.

A systematic review found varying effects of providing tobacco cessation therapy parallel to the treatment of dependence syndromes [13]. Although most patients with opioid dependence smoke tobacco, few are offered smoking cessation treatment [14]. Among patients with opioid use disorder who were offered smoking cessation interventions, previous studies have generally not succeeded in smoking cessation [15].

On the other hand, combined counselling and nicotine replacement therapy (NRT) may, to some extent, improve the smoking cessation rate [13]. For patients receiving methadone as OAT medication, combined behavioural therapy and NRT have provided higher smoking cessation rates [16]. Financial incentives and NRT show promising results among patients with opioid use disorders [15].

In the general population, incentives boost cessation rates [17]. Motivational interviewing, including sessions up to 20 min, seems to aid in smoking cessation [18]. Behavioural interventions alone or in combination with pharmacotherapy increase quit rates [19]. The combination of behavioural intervention and provision of cessation medication increased quit rates in newly diagnosed cancer patients [20]. Among OAT patients on buprenorphine combination treatment for smoking cessation increased quit attempts and motivation to stop smoking, as well [21].

No trial has yet tested the effects of a combined smoking cessation intervention integrated into the OAT on the smoking pattern, pulmonary health, physical fitness, and mental health. A recently conducted pilot study with a similar intervention indicates promising results (submitted). We will thus conduct a multicentre randomised controlled trial to investigate the effect of combined smoking cessation intervention administered weekly for up to 24 weeks on smoking patterns, psychological well-being, and physical tests.

## Objectives {7}

This paper presents the protocol of the ATLAS4LAR smoking cessation intervention. The primary objective is to assess the effect of integrating smoking cessation therapy at OAT clinics compared with standard OAT (control arm) on the self-reported number of cigarettes smoked and carbon monoxide levels in the exhaled air.

The secondary objectives are to investigate the change in psychological distress, impact of smoking cessation on inflammation, physical tests, and assessment of changes in quality of life, fatigue, and psychological well-being in the trial arms.

## Trial design {8}

This study is designed as a multicentre individually randomised controlled superiority trial with two parallel groups and an allocation ratio of 1:1.

## Methods: participants, interventions, and outcomes

### Study setting {9}

The target group will be patients with severe opioid dependence receiving OAT from outpatient clinics in the Norwegian cities Bergen and Stavanger, who are smoking tobacco. The Department of Addiction Medicine at Haukeland University Hospital in Bergen and the Department of Substance Abuse and Addiction Treatment, Stavanger University Hospital in Stavanger, have adopted an integrated treatment and care model for patients receiving OAT. In Bergen, OAT outpatient clinics have been established in each district where the patients are followed up by health and social workers on a nearly daily basis with observed intake of the OAT medications [2]. This group of patients has a large morbidity burden and has a limited degree of being able to access other standard health care. Each of the OAT outpatient clinics is staffed by a consultant and a physician specialising in addiction medicine in addition to nurses, social workers, and several of the clinics also being staffed by a psychologist. OAT Stavanger has a relatively similar structure. The treatment model in Bergen and Stavanger is an excellent platform to test out integration of additional intervention for patients receiving OAT aiming to improve health and life span of a vulnerable group, and at the same time gathering knowledge which traditionally have been very difficult to obtain.

### Eligibility criteria {10}

For the randomised trial, inclusion will be based on the following criteria:

- Receiving OAT from an included outpatient clinic with weekly follow-up
- Smoking at least one cigarette per day or seven cigarettes per week
- Obtaining informed consent

The following exclusion criteria will be used:

- Allergies or prior anaphylactic reactions to medication used
- Smoking less than three times a week
- Already using smoking cessation medications

### Who will take informed consent? {26a}

Research nurses at the involved OAT clinics will recruit patients and obtain informed consent.

### Additional consent provisions for collection and use of participant data and biological specimens {26b}

The consent forms specifically state that the collection of blood samples and physical tests are a part of the study and that participation is voluntary. Refusal to participate will involve no penalty or loss of benefits, and the subject may discontinue participation at any time without penalty or loss of benefits to which the subject is otherwise entitled. The specimens will only be used for research that is described in the protocol and consent. All biological specimens will be destroyed at the end of the study or at a specified time (e.g. after analysis). Patients are, in addition, asked to consent to the collection and storage of their specimens in a biobank connected to Bergen Addiction Research Group which is administered by the Department of Addiction Medicine at the Haukeland University Hospital in Bergen, Norway.

### Interventions

#### Explanation for the choice of comparators {6b}

Comparators and participants in the intervention group are recruited from the same involved OAT clinics, to reduce selection bias.

#### Intervention description {11a}

Participants randomised to the intervention arm will be assessed at baseline (Table 1) and offered a weekly smoking cessation intervention consisting of behavioural interventions for smoking cessation and prescription-free nicotine replacement products in addition to standard opioid replacement therapy.

**Table 1:** Protocol schedule outlining follow-up visits and assessments at each visit

Time point	Study period					
	Enrolment	Allocation	Post-allocation			Follow-up
	−1	0	1–15	16 (12–20)	17–24	34–54
**Research nurse assessment**	X			X		X
**Informed consent**	X					
**Eligibility assessment**	X					
**Randomisation**		x				
**Weekly follow-up by OAT staff (intervention group)**			x		x	
**Clinical assessment**	X			X		X
**CO-measurement**	X			X		X
**Physical fitn. (4-min step-test)**	X			X		X
Full blood count, CRP*	X			X		X
**SCL-10 (mental health)**	X			X		X
**FSS-3 (fatigue symptoms)**	X			X		X
**EQ-5D-5L (quality of life)**	X			X		X

*Additional tests will be taken on clinical indication. Time points indicate weeks from initiation of intervention

The behavioural intervention is based on motivational interviewing and psychoeducational techniques and will be delivered weekly during the intervention.

**Fig. 1:**
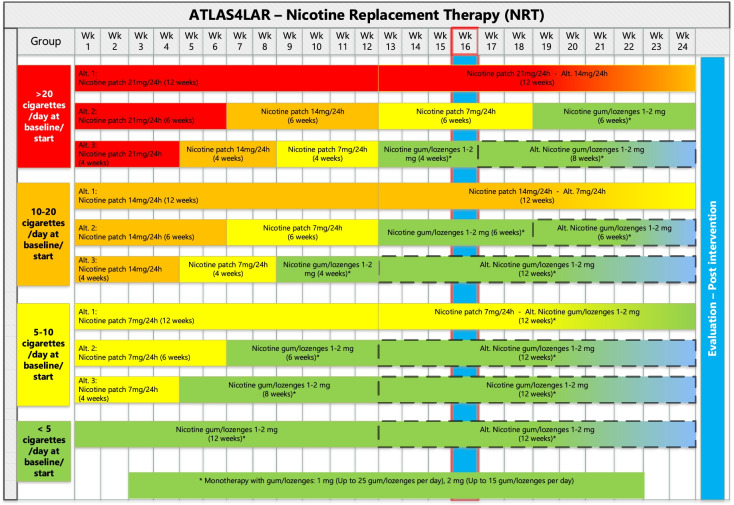
Timeline for nicotine replacement therapy component of the smoking cessation intervention. Remarks: The blue columns indicate evaluation points

In addition to the behavioural intervention, participants will have the opportunity to pick up prescription-free medications at the weekly appointments. Available medications are as follows: nicotine patches (21 mg/24 h, 14 mg/24 h, or 7 mg/24 h) and nicotine lozenges or chewing gum (both in 1 mg or 2 mg per units). Figure 1 and Table 2 give the exact dosing of the medication.

**Table 2:** Dosing table for nicotine chewing gum and lozenges

	Recommended daily dose	Maximum daily dose
**Patch and 2 mg chewing gum combined**	1 patch 6 chewing gums	1 patch 24 chewing gums
**Patch and 1 mg lozenges combined**	1 patch 6 lozenges	1 patch 24 lozenges
**Patch and 2 mg lozenges combined**	1 patch 6 lozenges	1 patch 15 lozenges
**Chewing gum (2 mg) monotherapy**	6 chewing gums	24 chewing gums
**Lozenges (1 mg) monotherapy**	6 lozenges	24 lozenges
**Lozenges (2 mg) monotherapy**	6 lozenges	15 lozenges

The table shows possible doses of nicotine chewing gum and lozenges as monotherapy or combined with nicotine patches. Patches are available in doses of 7 mg, 14 mg, or 21 mg per 24 h

If participants have managed to reduce the number of smoked cigarettes per day by at least 50 % at week 16, they may extend the intervention for another 8 weeks to attempt smoking cessation.

Participants randomised to the control arm will receive standard OAT treatment. At baseline, they will complete the assessment also offered to the intervention group (Table 1).

#### Criteria for discontinuing or modifying allocated interventions {11b}

Participants will be allowed to change their choice of NRT medication, within the limits described in Fig. 1, during the trial.

#### Strategies to improve adherence to interventions {11c}

Intervention will be linked to other treatment follow-up to improve adherence.

#### Relevant concomitant care permitted or prohibited during the trial {11d}

There are no restrictions.

#### Provisions for post-trial care {30}

Following the trial participants will be offered yearly health assessments at their local OAT clinic.

### Outcomes {12}

Primary outcome measures are**:**

- Proportions of participants achieving smoking cessation verified by carbon monoxide (CO) levels below six parts per million (ppm) at the end of the intervention or at least a 50% reduction in number of cigarettes smoked by week 16 of the intervention period (range 12–16 weeks after intervention initiation).

Secondary outcome measures are measured at the same time point as the primary outcome and include:

- Number of cigarettes smoked and CO levels (in ppm) in exhaled air.
- Biochemical indicators of inflammation
- All: C-reactive protein in serum and total leukocyte count in blood
- Sub-group (*n* = 60): IFN-gamma, IL-1beta, IL-1RA, IL-6, IL-8, IL-10, IL-17A, MCP-1, TNF-alfa measured in dried blood spots
- Changes in psychological well-being will be assessed with the Norwegian validated translation ten-item version of Hopkins Symptom Checklist (SCL-10) [22], compared to baseline.
- Physical fitness assessed with 4-min step-test measuring number of steps climbed in period [23].
- Changes in quality of life will be assessed with EuroQoL EQ-5D-5L [24], compared to baseline.
- Changes in fatigue will be assessed with the Fatigue Symptom Scale (FSS-3) [25], compared to baseline.
- Symptoms of dyspnoea will be assessed with the modified Medical Research Council dyspnoea scale (mMRC) [26].
- Physical activity is recorded with the International Physical Activity Questionnaire (IPAQ) [27].

### Participant timeline {13}

Participant timeline is presented in Table 1.

### Sample size {14}

Smoking cessation: We expect, based on a pilot study (not yet published), that the intervention will increase the proportion of patients not smoking by 11%.

The power calculation is based on the following assumptions:

- The power is set at 80% with a two-sided alpha (*α*) error of 5%.
- Comparison of proportion of smokers at baseline and at 16 (12–16) weeks.
- Up to 20% lost to follow-up at 16 weeks after treatment.
- Equal proportions between the groups, 11% higher rates of smoking cessation compared to standard treatment and 4% effect of comparison on smoking cessation in control arm.

Based on these assumptions, 133 persons are required in intervention arm and 133 persons in the control arm (statistical power calculations in Stata SE 17.0).

We also expect that the intervention will reduce the number of cigarettes smoked after the intervention by 30% if the patients do not achieve smoking cessation. Similarly, an additional power calculation is based on the following assumptions:

- The power is set at 90% with a two-sided alpha (*α*) error of 5%.
- Comparison of proportion of smokers at baseline and at 16 (12–16) weeks.
- Up to 20% lost to follow-up at 12 weeks after treatment.
- Equal proportions between the groups, 30% reduction in cigarettes smoked compared to standard treatment in control arm.

Based on these assumptions, 133 persons are required in intervention arm and 133 persons in the control arm (statistical power calculations in Stata SE 17.0).

### Recruitment {15}

All patients receiving OAT from included clinics will be considered the reference target population. As part of an annual health assessment linked to the ATLAS4LAR project [15], patients will be informed about the study and asked for consent to participation. All patients in target population will be offered annual health assessment and study participation. For those giving informed consent, an extended clinical assessment will be offered and those fulfilling inclusion and not exclusion criteria will be randomised for the study.

## Assignment of interventions: allocation

### Sequence generation {16a}

We will use computer-generated block randomisation with a 1:1 ratio using blocks of eight to ensure relatively similar distribution between both arms throughout different time periods of the trial. Randomisation will be electronically registered. The randomisation will be stratified by site (site 1: Bergen and site 2: Stavanger).

### Concealment mechanism {16b}

Once all the eligibility criteria are fulfilled and consent is obtained, a unique patient identifier number will be entered into the randomisation spreadsheet generating the allocation to intervention or control arm.

### Implementation {16c}

The allocation sequence is generated using a randomisation algorithm made through Stata that is linked to an electronic number for each patient in a software for digital patient involvement. Research nurses will enrol and assign participants.

## Assignment of interventions: blinding

### Who will be blinded {17a}

Even though complete blinding is regarded as difficult and infeasible. Patients will be informed of the follow-up they will receive, but not on other follow-up alternatives that are used or the exact hypotheses for the study. The outcomes assessor will be blinded.

### Procedure for unblinding if needed {17b}

Not applicable, as study nurses know patient assignment.

## Data collection and management

### Plans for assessment and collection of outcomes {18a}

Data collection and follow-up will be given in line with Table 1 and Fig. 1.

The blood samples for the primary outcome measures will be collected at the OAT clinics. Outcome measures will be measured/collected at the OAT clinics by research nurses through a structured interview for both participants randomised to standard and integrated treatment. Following the intervention period, participants will receive a yearly health assessment as part of their standard OAT.

### Plans to promote participant retention and complete follow-up {18b}

All participants who agree to participate in the trial will be shown appreciation for their willingness to participate. Undergoing the assessments, the participants will be able to establish a relationship to research staff, which will provide a good opportunity for information and communication about issues concerning the trial. We will strive to give the trial participation experience a sense of being part of a community of patients taking part in research. Patients who discontinue will be offered a consultation without obligations with the study nurse to explore reasons for discontinuing. If participants discontinue the trial, they will be offered a new assessment at the time of the yearly health assessment. As the trial is integrated into ordinary OAT, patients will receive weekly follow-up and reminders of appointments with research staff.

### Data management {19}

All data will be collected using electronic data collection software (Checkware®) under research nurses’ supervision. Data is stored on a secure research server provided by the University Hospital of Bergen. All the clinical data, including information regarding OAT, OAT medication, substance use, and possible comorbid clinical conditions, will be collected from the electronic medical record.

### Confidentiality {27}

All personal data is stored on a secure, access-restricted research server. The senior investigators LTF and JHV will import data from the collection software (Checkware®) and from the electronic medical record to a common file using each participant’s Norwegian personal identification number. Each participant is then given a computer-generated identification number for further analysis. Only anonymised data will be published.

Research nurses use paper forms for collecting the data during the trial and before data is plotted into the collection software. Appointments are made using the medical record system. The research nurses store all paper forms that may be connected to a participant in a locked file in a room with restricted access.

For documentation and follow-up purposes, data will be stored until the end of the project on the 31st of December 2029 and then deleted.

### Plans for collection, laboratory evaluation and storage of biological specimens for genetic or molecular analysis in this trial/future use {33}

Biological specimens collected in this trial are blood samples. These will be analysed and only the results will be stored. For patients who consent to collection of specimens for the biobank, the specimens will be stored in the biobank (ethical approval 2016/1080/REK vest). According to the ethical approval, use of the samples in the biobank for future projects requires new ethical review.

## Statistical methods

### Statistical methods for primary and secondary outcomes {20a}

A detailed plan for analysis is provided in Table 3. Analysis methods will follow the CONSORT and SPIRIT guidelines [28–30]. All tests will be two-sided. Descriptive results and efficacy estimates will be presented with 95% confidence intervals. The statistical significance is set at *p* < 0.05. Potential confounders may be considered for adjustment if they are imbalanced at baseline (with assumed meaningful differences). Variables will be summarised as percentages and continuous variables as medians with interquartile ranges or means with standard deviation for variables with a Gaussian distribution. The main outcomes will be analysed with generalised linear models (Gaussian distribution).

**Table 3:** Analysis plan

Variable/outcome	Hypothesis	Outcome measure	Method of analysis
**1. Primary**			
a. Proportion of patients smoking	Intervention improves smoking cessation rates from baseline to 16 weeks	Carbon monoxide in ppm in exhaled air	Chi-squared test
b. Proportion achieving at least 50 % reduction in number of cigarettes smoked	Intervention reduces number of cigarettes smoked from baseline to 16 weeks	Self-reported daily number of cigarettes smoked	Chi-squared test
**2. Secondary**			
Number of cigarettes smoked	Reduction in number of cigarettes	Self-reported daily number of cigarettes smoked	*t*-test and regression methods with secondary outcomes as dependent variable adjusted for variables defined under additional analysis
Carbon monoxide in exhaled air	Reduced CO levels	Carbon monoxide in ppm in exhaled air	
C-reactive protein	Reduced levels	CRP in mg/L	
Leucocyte count	Levels within reference limit	Leucocyte count in 10^9^/L	
Psychological well-being	Increased score	Hopkins Symptom Checklist (SCL-10)	
Physical fitness	Increased score	4-min step test, number of steps	
Quality of life	Increased score	EuroQoL EQ-5D-5L-questionaire	
Fatigue	Less Fatigue	Fatigue Symptom Scale (FSS-3)	
Dyspnoea	Less after intervention	Modified Medical Research Council (mMRC)-scale	
Physical activity	Increased	Physical Activity Questionnaire (IPAQ)	
**3. Additional analysis**			
OAT-medication	Choice of OAT-medication impacts primary outcome		Regression methods with OAT medication as categorical co-variate.
OAT-medication doses	Higher doses inhibits smoking cessation		Regression methods with OAT-doses as independent variable
Adjusted for age	Co-variates impact the outcomes of the trial		Regression methods with appropriate interaction term
Adjusted for sex			
Adjusted for i.v. drug use			
Adjusted for known COPD			
Impact of number of cigarettes smoked on secondary outcomes	Fewer cigarettes smoked results in improved secondary outcomes		Regression methods with secondary outcome as dependent variable and number of cigarettes smoked as independent variable

*Important remarks*: In all analyses will be expressed as coefficient, standard errors, corresponding 95%, and associated *p*-values

Goodness-of-fit will be assessed by examining the residuals for model assumptions and chi-squared test of goodness-of-fit

Bonferroni method will be used to correct for multiple testing

### Interim analyses {21b}

Throughout the study period, there will be weekly meeting between the study nurses and the investigators. The meeting will consider progression of the trial and adverse events. The principal investigator LTF will make the final decision to terminate the trial.

### Methods for additional analyses (e.g. subgroup analyses) {20b}

Methods for additional analyses are presented in Table 3.

### Methods in analysis to handle protocol non-adherence and any statistical methods to handle missing data {20c}

We will use an intention to treat analysis strategy to handle protocol non-adherence. Missing data will be considered, and appropriate imputations based on pre-defined assumptions will be done when necessary (as described in detailed plan of analysis).

### Plans to give access to the full protocol, participant-level data, and statistical code {31c}

Upon request, the access to the full protocol, anonymised participant-level dataset, and statistical code is granted.

## Oversight and monitoring

### Composition of the coordinating centre and trial steering committee {5d}

Research nurses and the primary investigator (medical doctor) will meet on a weekly basis, during the study. The clinical team, including research nurse, at each OAT clinic will meet on a daily basis.

Once a week the principal investigator, other investigators, research nurses, and user representatives will meet (study coordination unit).

### Composition of the data monitoring committee, its role, and reporting structure {21a}

An external data monitoring committee is not needed as this study is a health services research evaluating the integration of a smoking cessation intervention. The medications used in the study are used according to the manufacturers’ recommendation, and evaluating the effect of the medications is not a primary objective outcome of this study but rather an integrated smoking cessation intervention package.

### Adverse event reporting and harms {22}

Those who participate in the study will be randomised to one of two different follow-up programmes. It is possible that the intervention will be inferior in outcomes compared to the standard treatment. Some might have allergies to components in the administered nicotine products, but severe allergies to these are rare, and patients with severe allergies who are vulnerable to negative reactions will be excluded from participation in this trial. For nicotine gum products hiccoughs, gastrointestinal disturbances, jaw pain, and oro-dental problems are the most frequently reported side effects [31]. Nicotine lozenges have been reported to cause hiccoughs, burning and smarting sensation in the mouth, sore throat, coughing, dry lips, and mouth ulcers, whereas skin sensitivity and local skin irritation is common among users of nicotine patches [31]. Adverse events or unintended effects may be reported to the research nurses at daily meetings in the OAT clinics. Patients reporting adverse events will be offered an evaluation by the research nurse or research physician. The study coordination unit will be responsible to ensure safety, adherence to the protocol, quality of the study, and ethical conduct. It is also possible that follow-up with motivational interviewing approach could be considered as time consuming and unwanted. The participants could at any time choose to abort this intervention.

### Frequency and plans for auditing trial conduct {23}

Bi-annual internal auditing of trial will be conducted.

### Plans for communicating important protocol amendments to relevant parties (e.g. trial participants, ethical committees) {25}

Any substantial modifications to the protocol, which may impact on the conduct of the study, will be reported to the regional ethical committee for renewed approval. Participants will be informed of the changes and the published protocol will be amended accordingly.

## Dissemination plans {31a}

Results of the trial will be published in peer-reviewed medical journals. We will submit abstracts to relevant national and international congresses. Participants and clinical staff at the participating OAT clinics will receive summaries of the outcomes.

## Discussion

This study will improve understanding of how a combined intervention aiming for smoking cessation over a period of up to 6 months could succeed in reducing smoking, and improve psychological well-being and physical tests. This could potentially reduce future morbidity and mortality among patients with opioid dependence receiving OAT.

Smoking cessation or reduction has been considered as infeasible for most patients receiving OAT [13]. Studies have investigated interventions to quit smoking among patients with opioid dependence [16, 21, 32], but few have investigated smoking cessation interventions integrated into an OAT setting. Although OAT patients are a hard-to-reach group of patients, a previous study has shown that integrating treatment into the ordinary OAT follow-up increases adherence and completion rates [33]. We believe this study will be able to answer some of the questions raised in a recent meta-analysis on reducing tobacco use in people experiencing homelessness [34], including increasing accessibility, long-time follow-up impact on mental health and substance use.

Our trial involves some limitations and several strengths. For the trial, it is difficult to ensure complete blinding. The study is funded from public sources ensuring independency. We also have a biologically verified primary outcome. Thus, substantial information biases are considered unlikely. The study is individually randomised minimising potential confounding. The study population receiving OAT will include a sufficient large study sample size to answer the primary objectives with high precision and is assumed to have adequate precision also for secondary objectives. In terms of safety, the trial is considered as a low-risk study. Our conventional trial design is less vulnerable to confounding from time trends than other designs such as a stepped-wedge design.

If the smoking cessation intervention integrated into OAT is superior to standard OAT in terms of assessed outcomes, this intervention could be considered for further scale-up.

## Trial status

Trial protocol version 2, 14 July 2022. Start of recruitment 19 April 2022. Estimated completion of recruitment 19 June 2022.

## Data Availability

Authors and persons mentioned under acknowledgements will have access to the final trial dataset.

## Abbreviations

COPD: Chronic obstructive pulmonary disease
OAT: Opioid agonist therapy
NRT: Nicotine replacement therapy
CO: Carbon monoxide
SCL-10: Hopkins Symptom Checklist
mMRC: Modified Medical Research Council dyspnoea scale
IPAQ: International Physical Activity Questionnaire
CRP: C-reactive protein
FSS-3: Fatigue Symptom Scale
PPM: Parts per million
